# Editorial: Drug Development for Parasite-Induced Diarrheal Diseases

**DOI:** 10.3389/fmicb.2017.00577

**Published:** 2017-04-04

**Authors:** Anjan Debnath, James H. McKerrow

**Affiliations:** Center for Discovery and Innovation in Parasitic Diseases, Skaggs School of Pharmacy and Pharmaceutical Sciences, University of CaliforniaSan Diego, La Jolla, CA, USA

**Keywords:** parasite, diarrhea, drug, *Entamoeba*, *Giardia*, *Cryptosporidium*, chemotherapy, protozoa

Diarrhea continues to be a major contributing factor to morbidity and mortality worldwide. It is the second leading cause of death in children under 5 years old. Three enteroparasites, *Entamoeba histolytica, Giardia lamblia*, and *Cryptosporidium parvum* are common infectious agents causing persistent diarrhea. The Global Burden of Disease Study (GBDS) found that amebiasis was responsible for more than 55,000 deaths and 2.2 million disability-adjusted life years (DALYs), and cryptosporidiosis accounted for about 100,000 deaths and 8.4 million DALYs in 2010 (Hotez, 2014). According to the WHO Foodborne Disease Burden Epidemiology Reference Group (FERG), giardiasis produced 171,100 DALYs in 2010 (Torgerson et al., 2015). Though giardiasis did not result in deaths, the economic burden of acute giardiasis in the US continues to be substantial. The annual cost involving the hospital based treatment for giardiasis is approximately $34 million (Collier et al., 2012). In addition, *E. histolytica, G. lamblia*, and *C. parvum* are considered to be bioterrorism threats by the National Institutes of Health (NIH) and Centers for Disease Control and Prevention (CDC).

In absence of vaccines to prevent these parasitic diseases, control relies on chemotherapy which is far from perfect and limited by adverse drug effects. For example, metronidazole is the most common drug used to treat invasive amebiasis and giardiasis, but treatment often leads to side effects, such as nausea, vomiting, diarrhea, or constipation. Moreover, potential resistance of *E. histolytica* to metronidazole is an increasing concern. Although millions of people are infected by *E. histolytica* each year, most individuals remain asymptomatic but may transmit amebiasis through fecal excretion of infective cysts. Therefore, an additional drug paromomycin is required following metronidazole treatment to eliminate cysts (Haque et al., 2003). However, treatment with two drugs for 20 days is long, burdensome, and reduces compliance. In spite of the reported efficacy of nitroimidazole drugs, treatment failures in giardiasis occur in up to 20% of cases and a recent report from the Hospital for Tropical Diseases, London found that the nitroimidazole therapy failure rate in giardiasis was 40.2% in 2013 (Nabarro et al., 2015). Nitazoxanide, the only treatment option for cryptosporidiosis, has an efficacy ranging from 56% in malnourished children to 80% in healthy adults and it is not effective for immunocompromised patients (Manjunatha et al., 2016). All these factors make the development of new antimicrobials to treat these infections a critical need to avert future outbreaks and deaths.

Recently, drug discovery efforts to treat parasite-induced diarrheal diseases are burgeoning. Researchers across the globe are working toward identifying better drug candidates for the treatment of these parasitic diseases. However, a comprehensive report of their endeavors encompassing these parasites is lacking. We launched this Research Topic to compile the recent progress made by the parasitology research community to develop effective drugs for the treatment of parasitic diarrheal diseases. It may also provide a one-stop resource for drug development research targeting enteric protozoan parasites. Seventy-three authors enriched the Research Topic with their contributions leading to 14 articles. These articles have been organized in an e-book and can be categorized in four sections.

The first section opens with a review of different heterocyclic compounds for their activity against intestinal protozoan parasites, and current knowledge of the drug targets and mechanism of action of some of the compounds (Azam et al.). This review not only provides a survey of drug discovery research on *E. histolytica, G. lamblia*, and *C. parvum* but also includes an update on drug development for other intestinal parasites such as microsporidia, *Isospora* and *Cyclospora*. A review article by Miyamoto and Eckmann highlights different assays used for drug development and detailed the implementation of both the activity-centered and target-centered strategies to develop new drug leads for cryptosporidiosis and giardiasis. The third article of this section focused on repurposing of an FDA-approved drug auranofin for the treatment of amebiasis and giardiasis and the description of *E. histolytica* and *G. lamblia* thioredoxin reductase as an attractive drug target (Andrade and Reed). Auranofin showed promising activity against multiple parasites and identifying a broad-spectrum antiparasitic agent may be a useful drug discovery strategy for rare and neglected parasitic diseases instead of applying “one bug-one drug” approach.

The second section includes five original research articles on drug discovery for giardiasis. The articles range from medicinal chemistry to mechanism of resistance studies and new drug target identification. In the medicinal chemistry research article, the authors synthesized 45 novel chalcone analogs with triazolyl-quinolone scaffold and tested their activity against *G. lamblia* (Bahadur et al.). Four compounds were particularly selective for *G. lamblia* trophozoites with IC_50_ better than metronidazole and less toxic against a human cell line. These new synthetic chalcone derivatives hold promise for future design of antigiardial compounds. Since resistance of *Giardia* to nitroimidazoles and albendazole is an emerging issue, it is imperative to understand the mechanisms involved in drug resistance in order to develop better treatment strategies. Arguello-Garcia et al. developed albendazole-resistant clones and compared the expression of different antioxidant enzymes in albendazole-sensitive and -resistant trophozoites. Albendazole was shown to induce the formation of reactive oxygen species causing DNA damage which led to apoptosis (Martinez-Espinosa et al.). In contrast, albendazole-resistant trophozoites over expressed antioxidant enzymes to detoxify reactive oxygen species. Two articles exploited *Giardia* metabolic enzymes for parasite specific drug development. An FAD-dependent glycerol-3-phosphate dehydrogenase of *G. lamblia* was characterized for activity and cellular localization (Lalle et al.). The authors validated the target by inhibiting *Giardia* glycerol-3-phosphate dehydrogenase activity chemically using an antitumor compound NBDHEX. Guo et al. considered *Giardia* acyl-CoA synthetase (GiACS) as potential drug target and recombinantly expressed GiACS1 and GiACS2. Chemical inhibition of GiACS1 and GiACS2 activity by an ACS inhibitor triascin C led to growth inhibition of *G. lamblia* trophozoites at low micromolar concentration. These studies pave the way for future *in vivo* studies and also the development of more potent inhibitors targeting these metabolic enzymes.

The year 2015 was particularly exciting for the parasitology community because 2015 Nobel Prize in Physiology or Medicine was awarded to three scientists who developed therapies against parasitic infections. These drugs had their origin in microbial and plant natural products. Our third section consists of four articles on drug discovery for amebiasis. One article (Mori et al.) was contributed by researchers of Kitasoto University and National Institute of Infectious Diseases, Japan and included Satoshi Omura as a co-author. Dr. Omura was awarded the 2015 Nobel Prize in Physiology or Medicine. This team screened the Kitasoto Natural Products Library and extracts of 9,173 fungal and actinomycete broths against two recombinant *E. histolytica* cysteine synthases. Identification of deacetylkinamycin C and nanomycin A as potent amebicidals may serve as a starting point for natural product based drug discovery for amebiasis. Identification of a protein that is involved in the virulence and regulation of different stages of life cycle of a parasite can aid in the development of new therapeutics. Singh et al. showed the involvement of *E. histolytica* heat shock protein 90 (Hsp90) in regulation of phagocytosis and encystation. This study together with a previous transcriptomic data of an increased expression of Hsp90 during excystation indicate that *E. histolytica* Hsp90 can be a promising therapeutic target. Shahinas et al. further validated the importance of parasite Hsp90 as a drug target by identifying five compounds that targeted *E. histolytica* Hsp90 and inhibited the growth of *E. histolytica* trophozoites. Though no *E. histolytica* clinical isolates with high levels of metronidazole resistance have been detected, reliance on a single drug for more than 50 years puts them at increased risk to develop drug resistance. Even a low level of metronidazole resistance in the culture led to several cellular changes in *E. histolytica* such as lower rates in growth, adhesion, phagocytosis, cytopathogenicity, and glucose consumption (Penuliar et al.). Transcriptional profiling identified genes related to oxidative stress, nucleotide binding, metabolism, and signal transduction that were differentially expressed in the resistant parasite (Penuliar et al.). This study should serve as a cautionary advice on the importance of continuing the search for an alternative drug.

The final section deals with an assay development for a *C. parvum* drug screen and identification of a new lead for the treatment of cryptosporidiosis. An improved and simplified qRT-PCR based assay was developed and made amenable to high-throughput screen for *C. parvum* (Zhang and Zhu). The assay was validated by screening a small set of compounds. Sonzogni-Desautels et al. identified oleylphosphocholine as a new drug lead for *C. parvum* infection. Oral treatment with 40 mg/kg/day of oleylphosphocholine cured the infection in immunocompromised mice and provided protection up to 30 days. It remains to be seen if similar protection can be achieved in immunocompetent mice. Still this is an encouraging finding for the development of future treatment in immunocompromised patients.

Together, this Research Topic provided the current status of drug discovery research in parasitic diarrheal diseases and covered a wide-spectrum of topics from traditional drug discovery method to target identification and mechanism of resistance.

## Author contributions

AD wrote the article and JM edited the article.

### Conflict of interest statement

The authors declare that the research was conducted in the absence of any commercial or financial relationships that could be construed as a potential conflict of interest.
